# Case Report: A case of intrahepatic papillary neoplasm of the bile duct with invasive carcinoma in a young patient and review of the literature

**DOI:** 10.3389/fonc.2025.1670234

**Published:** 2025-11-26

**Authors:** Linhui Hu, Hua Wan, Yuan Hong, Li Liang, Yan Wang

**Affiliations:** 1 Department of Infectious Disease, Peking University First Hospital, Beijing, China; 2 Department of Pathology, Peking University First Hospital, Beijing, China

**Keywords:** intrahepatic papillary neoplasm of the bile duct, diagnosis, prognosis, fever, carcinoma

## Abstract

**Introduction:**

Intrahepatic papillary neoplasm of the bile duct (IPNB) with invasive carcinoma is a rare cholangiocarcinoma with the most frequent site of origin being intrahepatic bile ducts. Given its rarity and non-specific clinical presentations, accurate diagnosis plagues clinicians to improve patient outcomes.

**Case presentation:**

We present a case of a 31-year-old male who initially exhibited fever. Routine ultrasonography and computed tomography (CT) revealed a large mass in the right liver lobe, suggesting a high likelihood of an infectious lesion. However, multidisciplinary discussion offered a variety of possible scenarios. The patient subsequently underwent an extended right hepatectomy (ERH), and histopathological examination suggested a intrahepatic IPNB with invasive adenocarcinoma.

**Clinical discussion:**

The diagnosis and management of IPNB remain challenging, particularly in patients who present with atypical clinical symptoms and lack significant abnormalities in laboratory tests. Early imaging plays a critical role in guiding the diagnostic process. However, comprehensive diagnostic speculations, clinical expertise, and even invasive detections are essential for establishing a definitive diagnosis and determining the appropriate treatment strategy. Given the potential to invasive carcinoma, early detection and resection are vital to improving prognosis.

## Introduction

Intraductal papillary neoplasm (IPNB) and its associated invasive carcinoma are biliary epithelial tumors with papillary or villous growths in the bile ducts, characterized by a fibrovascular core in the dilated bile ducts, partially combined with mucus secretion (1). IPNB is mainly found in the intrahepatic and extrahepatic bile ducts and can therefore be divided into intrahepatic intraductal papillary neoplasms (I-IPNB) and extrahepatic intraductal papillary neoplasms (E-IPNB) (1). Hepatic bile duct stones and Treponema pallidum infection are currently considered to be associated with the etiology of IPNB (2). The clinical manifestations of IPNB are mostly non-specific (3). Early and radical resection is an optimal option in patients with imaging diagnoses of IPNB to achieve a satisfactory outcome (4). Due to the low incidence, its etiology, clinical manifestations, diagnostic methods, and histopathological features have not been fully determined.

This report describes a 31-year-old man with recurrent fever who was ultimately diagnosed with I-IPNB associated with invasive carcinoma.

## Presentation of case

A 31-year-old male with a history of hepatic cysts and hepatic cystectomy in 2007 was admitted due to intermittent fever for 2 months, with a maximum temperature of 39.9 °C. No accompanying symptoms, such as cough, sputum, abdominal pain, diarrhea, jaundice, and abdominal discomfort, were presented. The abdominal contrast-enhanced computed tomography (CT) results on October 27, 2023, revealed a huge lesion (11cm×10.6cm×12.1cm) in the right liver lobe with septations, calcifications, and heterogeneous enhancement.

**Figure 1:**
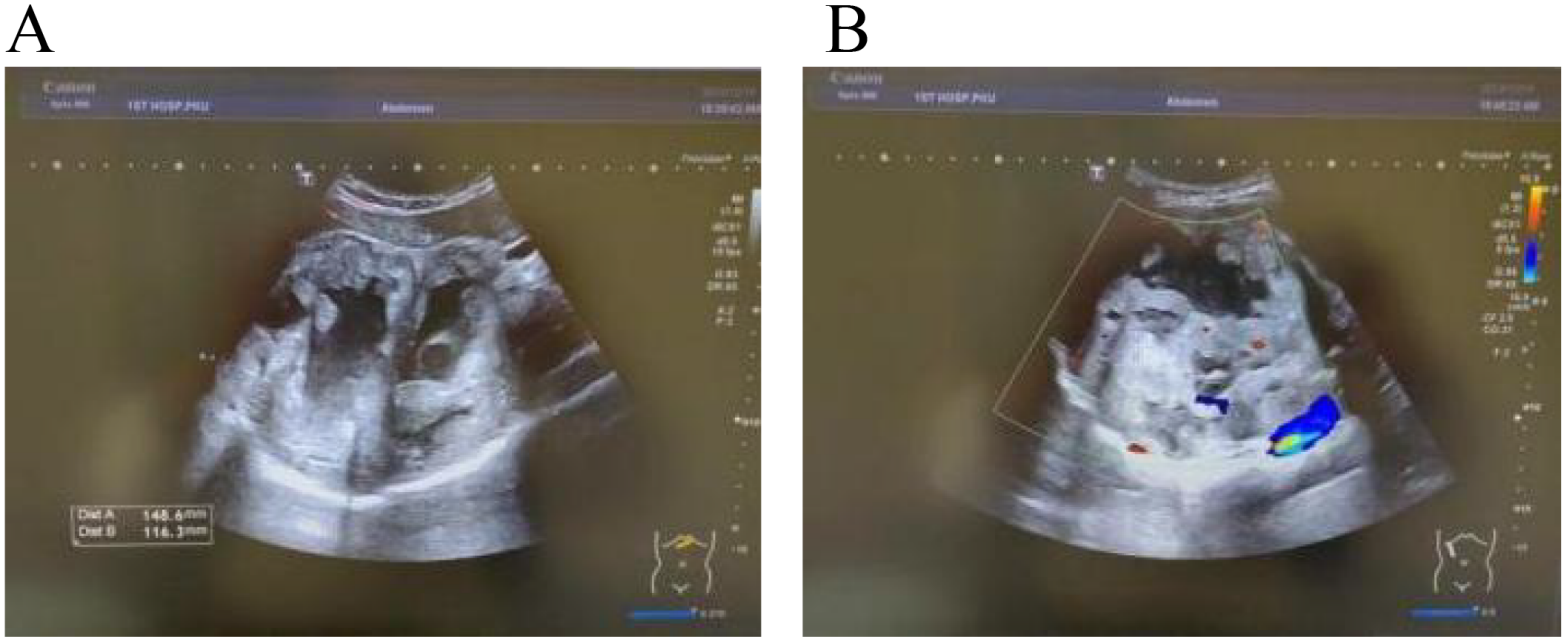
Ultrasound images. In **(A, B)** a heterogeneous hypoechoic area measuring 14.9×11.6 cm is visible in the right lobe of the liver, with unclear boundaries. Multiple anechoic areas are present within it, and no dilation of the intrahepatic or extrahepatic bile ducts is observed.

**Figure 2:**
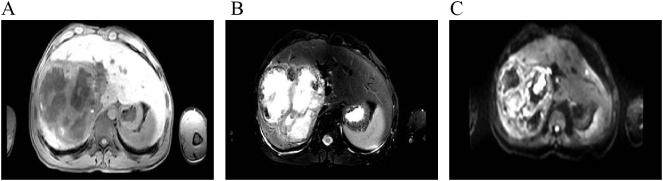
Contrast-enhanced abdominal MRI. **(A–C)** show T1-weighted imaging (T1WI), fsT2WI, and diffusion-weighted imaging (DWI), respectively. A large multiloculated cystic-solid mass is noted in the right hepatic lobe, appearing hypointense on T1WI and heterogeneously hyperintense on fat-saturated T2WI, with mildly hyperintense signal on DWI. The lesion measures approximately 11.6 cm × 11.3 cm × 11.6 cm. Contrast-enhanced imaging shows mild enhancement of the peripheral rim and internal septa. Fluid components exhibit hyperintensity on fs T2WI, and nodular or flake-like projections are visible along the inner wall of some cystic portions.

**Figure 3:**
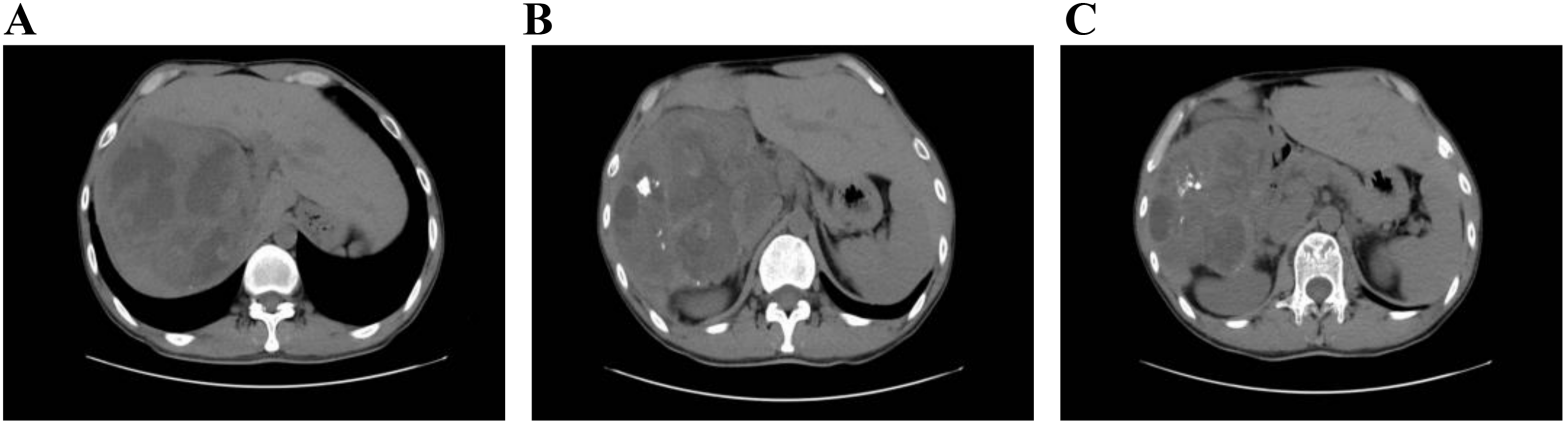
Abdominal and pelvic contrast-enhanced CT. In **(A–C)** the liver is normal in size and morphology. A large cystic-solid mass in the inferior right hepatic lobe contains multiple calcifications and measures approximately 13.4 cm × 12.6 cm × 12.1 cm. The lesion is well circumscribed and supplied by a branch of the hepatic artery. On multiphase contrast-enhanced imaging, the periphery and septa of the mass show enhancement, while internal low-density areas remain non-enhancing. No intra- or extrahepatic biliary dilatation is observed.

After admission, abdominal ultrasound (**Figures 1A, B**) results showed a heterogeneous hypoechoic area in the right lobe of the liver (14.9×11.6 cm) with unclear boundaries, and multiple internal anechoic areas. No dilation of the intra- or extrahepatic bile ducts was observed. The epigastric enhanced magnetic resonance imaging (MRI, **Figures 2A–C**) (2023-12-10) result showed a huge multicompartmental cystic solid lesion (11.6×11.3×11.6cm) in the right lobe of the liver with clear borders, and mild enhancement of the margins and internal septum, and a liquid fat suppression T2-weighted imaging (fsT2WI) high signal was seen in its interior. Abdominal and pelvic contrast-enhanced CT (**Figures 3A–C**) results showed that a large cystic and solid lesion was detected in the lower part of the right lobe, measuring approximately 13.4×12.6×12.1 cm with clear margins. The examinations of the blood routine and biochemistry showed normal findings. The inflammatory markers were elevated (C-reactive protein: 12.51 mg/L, erythrocyte sedimentation rate: 48 mm/1 h, interleukin 6: 7.15 pg/mL, procalcitonin: 0.06 ng/mL). However, liver and kidney functions as well as tumor markers were within the normal range. Antibodies to hepatitis A, B, C, D, and E viruses, cytomegalovirus/Epstein–Barr virus, Leishmania donovani, hepatic echinococcosis, Borrelia burgdorferi, antinuclear antibodies, antibody to autoimmune liver diseases, serum copper blue protein, stool search for amoebic trophozoites, and encapsulated bodies were normal.

**Figure 4:**
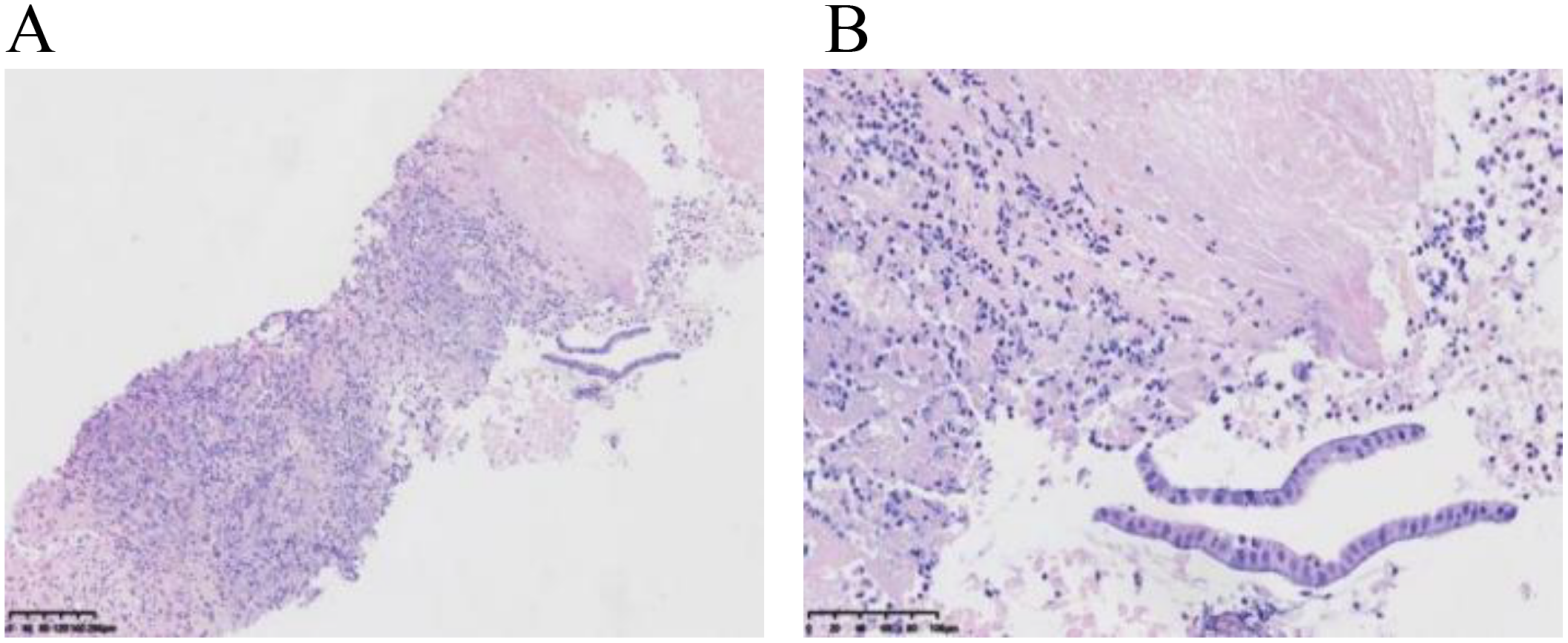
Pathology of liver puncture biopsy. **(A)** shows degenerative necrosis, infiltration of neutrophils and eosinophils, and a small amount of cuboidal epithelium in the biopsy tissue. **(B)** reveals a small amount of cuboidal epithelium with enlarged nuclei and slightly darker chromatin staining, with no granulomatous lesions observed.

Our multidisciplinary consultation suggested suspected diagnoses including biliary cystadenocarcinoma, chronic liver abscess, or atypical hepatic echinococcosis. A liver biopsy was required to collect liver tissue for pathological examination, bacterial culture, fungal culture, and biochemistry tests. Thereafter, a pathological report of liver biopsy (**Figures 4A, B**) indicated degenerated necrotic tissue with a large number of neutrophils and eosinophilic infiltration, and a minority of free cuboidal epithelium.

**Figure 5:**
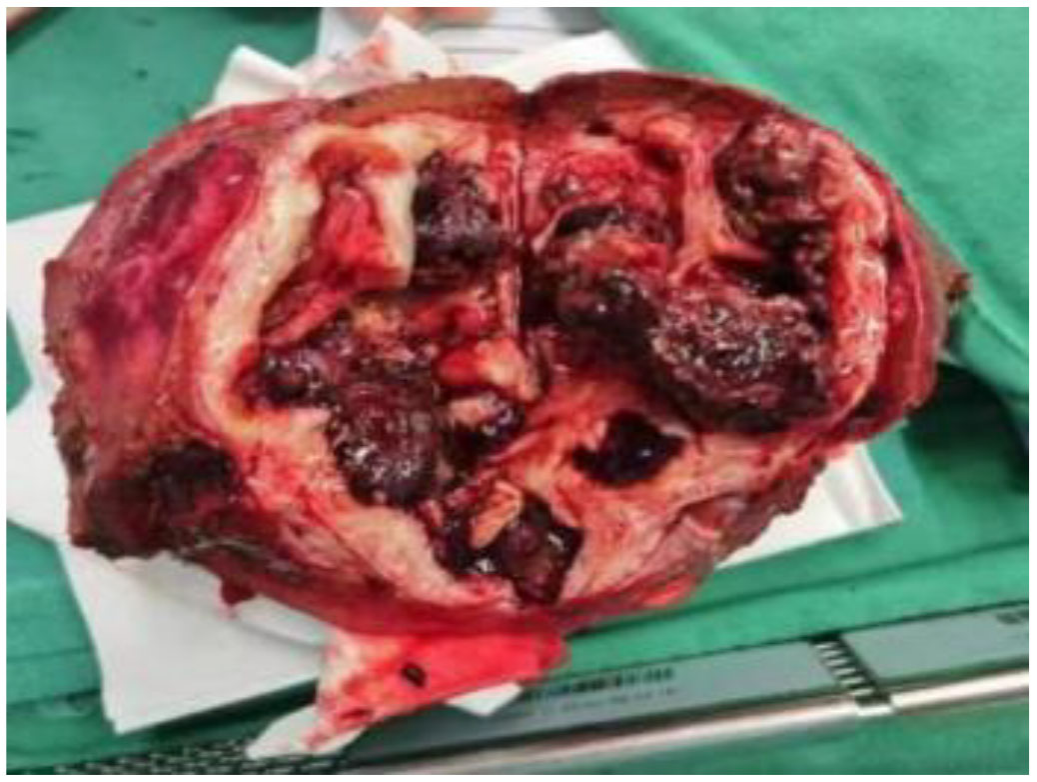
Surgical excision of postoperative tissue.

**Figure 6:**
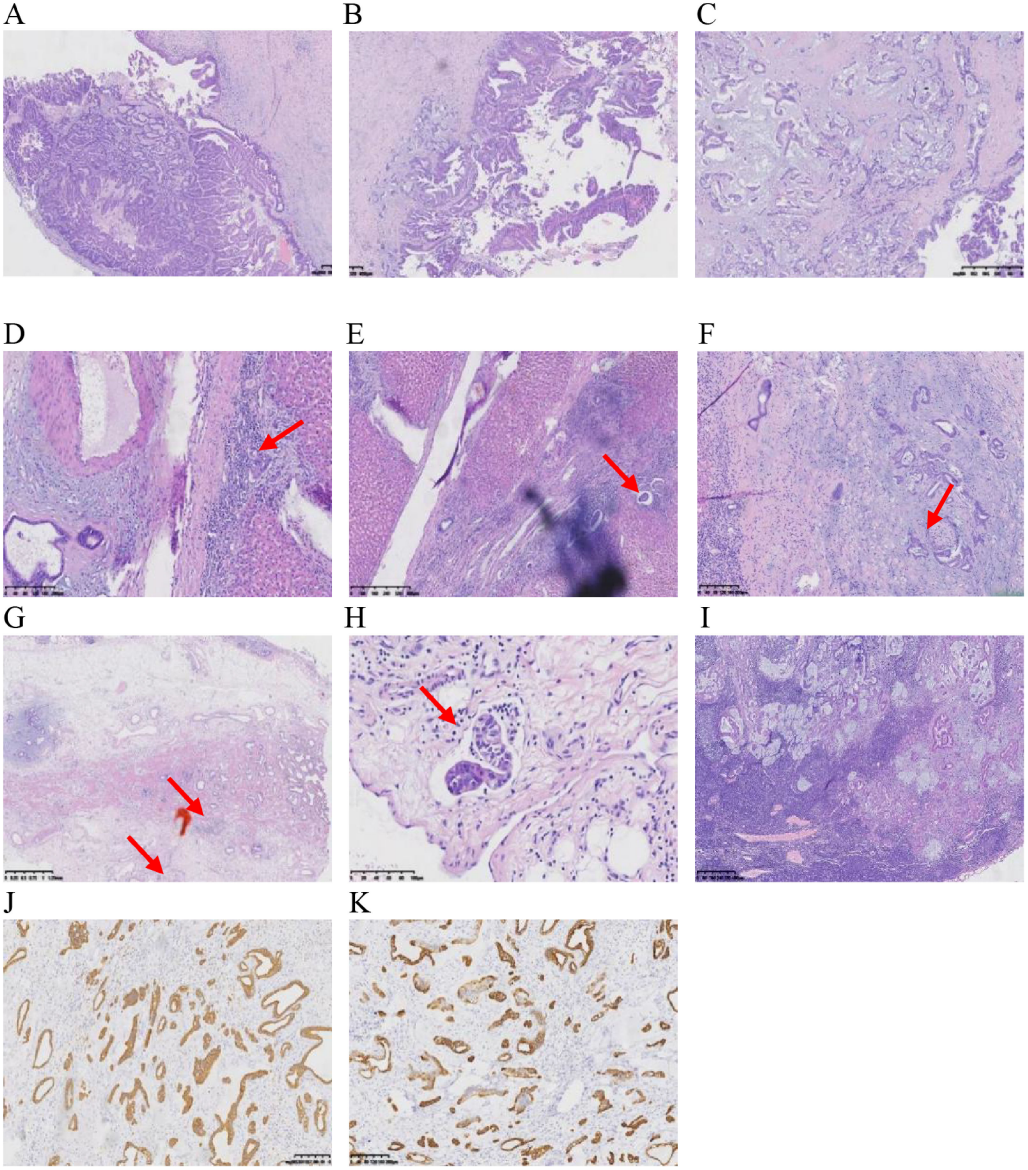
**(A, B)** show cystic solid area: papillary/villous, complex structure, invasive carcinoma, and predominantly bile duct type. **(C)** shows solid area structurally complex, invasive carcinoma with adenocarcinoma with mucus production. **(D)** shows peripheral hepatic tissue infiltration. **(E)** shows peripheral hepatic tissue choroidal carcinoma embolus. **(F)** shows nerve invasion of peripheral hepatic tissue. **(G, H)** show invasion of the outer membrane layer of the gallbladder wall, muscularis propria, and chorioallantoic embolus. **(I)** shows 1 hilar lymph node with cancer metastasis. **(J, K)** show the immunohistochemistry results, with positive staining for CK19 and CK7.

In anticipation of definitive surgical treatment, the patient ultimately underwent an extended right hepatectomy (ERH) at our center (**Figure 5**). The pathological examination revealed a large cystic and solid nodule (14×12×8 cm)with intrahepatic bile duct dilation, papillary hyperplasia of the biliary epithelium, and moderate to severe cellular atypia. Surrounding tumor cells exhibited glandular, band-like and nest-like infiltration, mucin production, and extensive necrosis (**Figures 6A–I**). Immunohistochemistry: CK7 (+++), CK20 (+), CK19 (+++), CDX-2 (weak +), GPC3 (−), hepatocyte (−), p53 (+), Ki67 (25%). Mismatch repair proteins: MLH1 (nuclear +), MSH2 (nuclear +), MSH6 (nuclear +), PMS2 (nuclear +) (**Figures 6J, K**). Based on these findings, the diagnosis was intrahepatic papillary neoplasm with infiltrative adenocarcinoma (pancreaticobiliary differentiation), with invasion of the gallbladder, vascular tumor thrombus, and lymph node metastasis.

Postoperative PET/CT showed that multiple lymph nodes in the right cardiophrenic angle and right parasternal region had increased glucose metabolism, likely representing metastasis. Within 1 year following discharge, the patient’s condition progressively worsened. Despite undergoing a series of chemotherapy treatments, the patient ultimately succumbed to the illness.

## Discussion

IPNB is more common in East Asian countries (including Japan, Korea, China, and Taiwan) than in Western countries. Its multistage carcinogenesis is regarded to be a mechanism by which chronic inflammation leads to biliary tract cancer (1). Risk factors for IPNB include hepatic bile duct stones, Chinese testicular schistosomiasis infection, primary sclerosing cholangitis, and biliary tract malformations (choledochal cysts and familial adenomatous polyposis or Gardner’s syndrome) (2). The demographic data indicate that patients are 35 to 91 years old with a slight male predominance (5). IPNB includes I-IPNB and E-IPNB, and of these, E-IPNB has a high incidence of malignancy (6). I-IPNB most often occurs in the left half of the liver as a multifocal papillary or cystic or cystic-solid lesion growing into the lumen. Our patient was a 31-year-old young male with a history of hepatic cysts and hepatic cystectomy. I-IPNB with invasive carcinoma was removed after right hemihepatectomy, but his prognosis was poor due to metastasis.

Based on the Japanese–Korean consensus, IPNB has been subdivided into two types: type 1 is the prototype of “classic IPNB”, and type 2 is the prototype of “so-called papillary or cholangiocarcinoma” (7). There are four pathological subtypes of IPNB: intestinal, gastric, pancreaticobiliary, and eosinophilic, with the intestinal type being more common in Asia and the pancreaticobiliary type being more common in the United States and Europe (8, 9). In the present case, the pathology suggested a pancreatobiliary type.

The clinical manifestations of patients with IPNB with invasive carcinoma are non-specific and depend mainly on the location of the tumor (3). The typical clinical manifestations are right upper abdominal pain, jaundice, fever, and acute cholangitis, but there are a certain number of asymptomatic cases (10). In terms of laboratory tests, patients with type 2 IPNBs have significantly higher levels of liver enzymes, bile enzymes, and tumor markers than patients with type 1 IPNBs. Preoperative diagnosis of IPNBs relies on imaging, which typically shows biliary dilatation, intraluminal mucus, and protruding into the lumen of the canaliculi. CT and MRI are the most commonly used imaging modalities, and the latter has the advantage of distinguishing benign from malignant lesions (11, 12). CT showed bile duct dilatation in 98.2% and an intraductal mass in 92.9% of cases, with IPNB enhancing to iso or hyper attenuation in the late arterial phase and becoming isoattenuating in the portal venous and delayed phases (10). On MRI scans, these structures are usually low signal on T1W1 and relatively high signal on T2W1 and DW1 (10). MRI combined with MR cholangiography reliably distinguishes invasive IPNB from intraepithelial neoplasia. Intraductal mass visualization, tumor diameter ≥2.5 cm, multifocality, bile duct wall thickening, and adjacent-organ invasion are independent imaging predictors of shorter recurrence-free survival (11). However, the lack of typical clinical manifestations, laboratory data, and imaging manifestations in this case poses a challenge to its diagnosis. In the setting of fever and compatible imaging features, a limited differential diagnosis for the hepatic lesions should be systematically considered.

1. IPNB

IPNB typically manifests as intermittent right-upper-quadrant pain, fever, cholangitis, or jaundice, but 12% of patients are asymptomatic (10). Serum carbohydrate antigen 19-9 (CA19-9) correlates with tumor burden yet lacks diagnostic specificity (10). Histological appearance varies with subtype, mucin production, atypia grade, extent of invasion, and biliary location (10). Definitive diagnosis of IPNB requires postoperative histopathology, so multimodal imaging should be used preoperatively to maximize diagnostic accuracy.

2. Mucinous cystic neoplasm (MCN) of the liver

MCN arises almost exclusively in the pancreatic body and tail of middle-aged women and invariably harbors ovarian type stroma (13). MCNs are mucin-containing mediastinal cystic tumors or columnar epithelium with ovarian-like mesenchyme between the endo-epithelial lining and the outer connective tissue peritoneum; MCNs usually have no upstream biliary dilatation (14, 15). The lesions are not connected to the bile ducts, and calcification of the cystic wall is usually seen (15). Mucinous cystadenocarcinoma, the malignant form of MCN, shows a higher rate of invasive carcinoma in men than in women, and EUS-guided fine-needle aspiration (EUS FNA) is recommended when imaging findings are equivocal (13).

3. Liver worm disease

Imaging mainly shows hepatic cystic space-occupying lesions, with solid or cystic solid masses of mixed density in the liver, and no abnormal enhancement, and some of them can be seen with characteristic small vesicles and calcification (16).

4. Bacterial liver abscess

Liver abscess is characterized by severe systemic toxicity and pronounced leukocytosis (17). Typical CT findings of liver abscesses include central liquefactive necrosis, the presence of an air-fluid level within the abscess cavity, and a surrounding “ring-target” sign (18).

5. Simple hepatic cysts

Simple hepatic cysts have thin and smooth cystic walls, no nodules within the lesion, no calcifications, homogeneous cystic fluid, and no communication with the intrahepatic bile ducts (11).

In patients with suspected IPNB on imaging, early surgery is desirable to achieve radical resection.

IPNB has different characteristics for different lesion sites (intrahepatic and extrahepatic) and tumor properties (dysplastic and invasive carcinoma). Surgical resection should be performed in patients without any evidence of distant metastases (19). If the intrahepatic lesion is too large to be completely resected, preoperative portal vein embolization may be performed to induce hypertrophy of the residual liver (20). Liver transplantation has been shown to result in good survival in cases of bilateral involvement or end-stage liver disease that cannot be partially resected (21). When major surgery is not required, adjuvant therapy includes chemotherapy, percutaneous transhepatic biliary drainage, percutaneous cholangioscopic laser ablation, and iridium-192 endoluminal therapy (22). In our patient, the gemcitabine and cisplatin regimen started immediately after major hepatectomy, but the prognosis was poor due to metastasis.

## Conclusion

In the present case, the patient was febrile yet liver function tests and serum tumor markers alpha fetoprotein (AFP), carcinoembryonic antigen (CEA), and CA19-9 were all within normal limits. Concurrently, no typical imaging findings, i.e., neither a solid mass component nor a hepatic bile duct stone, was observed, and no dilation of the intrahepatic or extrahepatic bile ducts was observed. After pathological diagnosis, it was confirmed as an invasive cancer associated with biliary papillomatosis. 17 years ago, the patient underwent resection of a right hepatic cyst, during which multiple septated lesions within the liver with thick walls and abundant blood flow signals were discovered. These septated lesions could progress with bile stasis and recurrent infections, probably leading to malignant transformation. The patient remained asymptomatic for 17 years after the hepatic cyst surgery and consequently did not adhere to regular follow-up. IPNB with characteristics of large cystic masses or liver abscesses are rare (23). This reminds us to remain vigilant with complex hepatic cysts with thick walls. This case highlights the importance of thorough preliminary imaging analysis, pathological diagnosis, and leveraging extensive clinical experience to guide assumptions and examinations, especially for patients with atypical presentations.

## Data Availability

The raw data supporting the conclusions of this article will be made available by the authors, without undue reservation.
